# Selective Vulnerability of Spinal and Cortical Motor Neuron Subpopulations in delta7 SMA Mice

**DOI:** 10.1371/journal.pone.0082654

**Published:** 2013-12-06

**Authors:** Paolo d’Errico, Marina Boido, Antonio Piras, Valeria Valsecchi, Elena De Amicis, Denise Locatelli, Silvia Capra, Francesco Vagni, Alessandro Vercelli, Giorgio Battaglia

**Affiliations:** 1 Molecular Neuroanatomy and Pathogenesis Unit, IRCCS Neurological Institute “C. Besta”, Milano, Italy; 2 Neuroscience Institute Cavalieri Ottolenghi (NICO), University of Torino, Orbassano (Torino), Italy; University of Turin, Italy

## Abstract

Loss of the survival motor neuron gene (*SMN1*) is responsible for spinal muscular atrophy (SMA), the most common inherited cause of infant mortality. Even though the SMA phenotype is traditionally considered as related to spinal motor neuron loss, it remains debated whether the specific targeting of motor neurons could represent the best therapeutic option for the disease. We here investigated, using stereological quantification methods, the spinal cord and cerebral motor cortex of ∆7 SMA mice during development, to verify extent and selectivity of motor neuron loss. We found progressive post-natal loss of spinal motor neurons, already at pre-symptomatic stages, and a higher vulnerability of motor neurons innervating proximal and axial muscles. Larger motor neurons decreased in the course of disease, either for selective loss or specific developmental impairment. We also found a selective reduction of layer V pyramidal neurons associated with layer V gliosis in the cerebral motor cortex. Our data indicate that in the ∆7 SMA model SMN loss is critical for the spinal cord, particularly for specific motor neuron pools. Neuronal loss, however, is not selective for lower motor neurons. These data further suggest that SMA pathogenesis is likely more complex than previously anticipated. The better knowledge of SMA models might be instrumental in shaping better therapeutic options for affected patients.

## Introduction

Spinal muscular atrophy (SMA) is an autosomal recessive neuromuscular disease. It is the most common inherited cause of infant mortality with an incidence of around 1 in 10000 live births and a carrier frequency of 1:31 [1]. The major anatomopathological hallmark of SMA consists in the degeneration of spinal motor neurons associated with a progressive atrophy of the limb and trunk muscles and, in the most severe cases, death. Based on the age of onset and disease severity, SMA has been classified into four main clinical types (I-IV) caused by homozygous loss or mutation of the Survival Motor Neuron 1 (SMN1) gene [2]. In humans, there are two *SMN* genes, the telomeric *SMN1* coding for an ubiquitous protein (full-length SMN or FL-SMN), and its centromeric homolog *SMN2* mostly generating a protein lacking exon 7 (Δ7-SMN), which is thought to be not functional or rapidly degraded [3-5]. The *SMN2* gene does, however, produce a 10% of FL-SMN so that high copy number of *SMN2* can dampen the clinical severity of SMA [6,7]. The 38 KDa FL-SMN protein is expressed in both the cytoplasm and the nucleus and plays a critical role in small nuclear ribonucleoproteins (snRNPs) assembly and pre-mRNAs maturation [8,9]. However, SMN also localizes in motor neuron axons *in vivo* [10,11] and a specific axonal role of the protein was proposed [12,13]. The role of SMN in axons received further support by the identification of an axonal form of SMN (a-SMN) [14], more selectively expressed in motor neuron axons and involved in axonogenesis. However, the molecular and cellular mechanisms by which *SMN1* gene mutations eventually lead to a selective failure of the neuromuscular unit remain unclear. 

Different mouse models of SMA were generated to understand disease pathogenesis [15-18]. Homozygous *Smn* gene deletion resulted in massive cell death before implantation [19], whereas engineering several copies of the human *SMN2* transgene on the *Smn*-null background prevented embryo-lethality and introducing additional mutated [20,21] or exon 7-deleted [17] human SMN1 cDNAs variously extended mice survival. Heterozygous Smn^+/-^ mice [22] or *Smn* ^2B/-^ mice harboring an allele mutated in the splicing enhancer region of exon 7 [15,23] displayed mild/ intermediate phenotypes.

The most frequently used model is the Δ7 SMA mouse (SMN2^+/+^; SMN Δ7^+/+^; Smn ^-/-^) [17], with a lifespan of about 2 weeks and early impairment of motor behavior correlated with motor neuron loss. These mice have been also widely used to test different therapeutic approaches, including viral-mediated gene delivery and anti-sense oligonucleotides up-regulating FL-SMN transcript levels [24-26]. Although different aspects of the Δ7 model have been analyzed in a number of studies [17,27], discrepancies still exist on the number and subgroups of spinal motor neurons affected during disease progression. Furthermore, analysis of brain morphology and evaluation of a possible vulnerability of the upper motor system have not been carried out in Δ7 mice.

In the present study, we have characterized the spinal cord and brain of Δ7 mice during development. We demonstrated through stereological analysis in the cervical spinal cord that motor neuron loss occurred progressively from early post-natal stages but not at pre-natal stages. In addition, motor neuron loss was more selective for larger motor neurons, particularly those innervating proximal and axial muscles. Finally, quantification of cell density in the motor cortex revealed a specific loss of layer V pyramidal neurons, indicating that neuronal degeneration was not restricted to lower motor neurons.

## Materials and Methods

### Ethic statement

All the procedures involving animals were performed in accordance with national (DL n. 116, G.U., Supp. 40, February 18, 1992) and European Community Council guidelines (EEC Council Directive 86/60, OJ L 358, 1, December 12, 1987, Guide for the Care and Use of Laboratory Animals, U.S. National Research Council, 1996). The experimental protocol was approved by the Ethics Committee of the “C. Besta” Neurological Institute and by the Italian Ministry of Health (protocol number: BT1/2012). Particular care was taken to minimize the number of animals, their discomfort and pain. 

### Experimental animals

The original breeding pairs of Δ7 mice were purchased from Jackson Laboratory (stock number 005025; Jackson Lab, Maine, USA). The colony was maintained by interbreeding carrier mice, and the offspring were genotyped by PCR assays on tail DNA according to the protocols provided by Jackson Laboratory. Mice had free access to food and water. Data were obtained from tissues harvested from *Smn* ^*-/-*^ (SMA) and *Smn* ^*+/+*^ (WT) mice sacrificed at embryonic stage 19 (E19), checked by vaginal plug examination, postnatal day 4 (P4, pre-symptomatic stage), P9 (fully-symptomatic) and P13 (terminal stage), considering P0 as the day of birth. 

### Tissue preparation

Pups were deeply anaesthetized by intraperitoneal injections of 4% chloral hydrate (10 ml/Kg) and trans-cardially perfused with 4% paraformaldehyde in phosphate buffer (0.1 M PB, pH 7.2). Brains were removed, weighed and immersed in fixative for 2 h at 4°C. Samples were transferred overnight into 30% sucrose in 0.1 M PB at 4°C for cryoprotection, embedded in medium (Killik; Bio-Optica, Milan, Italy) and cut with a cryostat (Microm HM 550; Thermo Fisher Scientific Inc, Waltham, MA, USA). P4 and P9 brains were cut in serial 20 μm-thick coronal sections and mounted onto gelatin-coated slides to be processed for immunostaining. E19, P4 and P13 cervical spinal cords were dissected out, embedded in warm 6% Agar (Sigma Aldrich, St. Louis, MO, USA), and cut on a vibratome (Leica VT1000S; Heidelberg, Germany) in serial coronal 25 μm-thick (for embryos) or 30 μm-thick (for pups) sections.

### Histology, immunohistochemistry and confocal imaging

For Nissl staining, brain and spinal cord sections were mounted on 2% gelatin-coated slides and air-dried overnight. Sections were then hydrated in distilled water, immersed in 0.1% Cresyl violet acetate or 0.1% thionine (Sigma Aldrich) and cover-slipped with Eukitt (Bioptica). For immunohistochemistry (IHC), unspecific binding sites were blocked with 5% bovine serum albumin (BSA), 1% Triton X-100 for 2 h at room temperature (RT), immersed in 3% hydrogen peroxide (H_2_O_2_) to remove the endogenous peroxidase activity, rinsed in phosphate buffer saline (PBS) and incubated with goat polyclonal anti-Choline Acetyl Transferase (ChAT) antibody (Millipore, Billerica, MA, USA: diluted 1:150) overnight at RT in a humidified chamber. After rinsing in PBS, sections were incubated with biotinylated donkey anti-goat IgG (Santa Cruz Biotechnology, Santa Cruz, CA, USA: diluted 1:200) for 2 h, rinsed in PBS and then incubated with ExtrAvidin-peroxidase (Sigma Aldrich: diluted 1:4000) for 1 h. All antibodies were diluted in 0.01M PBS, 3% BSA, 0.5% Triton X-100. Peroxidase staining was obtained by incubating the sections in 0.075% 3,3’-diaminobenzidine tetrahydrochloride (DAB; Sigma Aldrich) and 0.002% H_2_O_2_ in 50 mM Tris-HCl pH 7.5. Section were air dried, dehydrated and coverslipped with DPX (BDH Prolabo, Dublin, Ireland). Adjacent sections were counterstained with 0.1% thionine or Cresyl violet acetate. For confocal imaging, free-floating sections were pre-treated with 4% sucrose in PBS and 100% cold methanol for 30 min, and aspecific binding sites were blocked with 10% normal goat serum (NGS) or normal donkey serum (NDS) in PBS with 0.2% Triton X-100 for 1 h at RT. Sections were then incubated overnight at 4°C with monoclonal mouse anti-Glial Fibrillary Acid Protein (GFAP: diluted 1:500) or monoclonal mouse anti-neurofilament-SMI32 (Covance, Emeryville, CA, USA: diluted 1:1000) antibodies. All primary antibodies were diluted in 1% NGS and 0.3% Triton X-100 in PBS. After rinsing in PBS, sections were incubated in biotinylated anti-mouse IgG (Jackson ImmunoResearch Laboratories; West Grove, PA, USA: diluted 1:200 in 1% NGS in PBS) for 1 h, followed by Cy2-conjugated Streptavidin (Jackson ImmunoResearch Laboratories: diluted 1:200 in 1% NGS in PBS). Finally, sections were incubated with the pan-neuronal fluorescent marker NeuroTrace^TM^ (Molecular Probes, OR, USA: diluted 1:200), mounted on slides and examined on a Radiance 2100 (Bio-Rad, Hercules, CA, USA) or Leica TCS SP5 confocal laser-scanning microscopes (Leica). 

### Spinal cord stereological counts

Stereological counts were performed at C3-C5 in post-natal mice and whole cervical levels in embryos, on both cresyl violet (for each genotype: E19, n=3; P4, n=3; and P13, n=5) and ChAT stained (P4, n=3; P13, n=4) sections. In Nissl-stained sections, the nucleoli of spinal motor neurons in ventral horns were counted at 40X magnification. Only neurons with an area ≥ 80 μm^2^ and located in a position congruent with that of motor neuron groups were counted [28]. All ChAT^+^ profiles located in the ventral horns of immunoreacted sections clearly displaying a nucleolus on the plane of the section were counted. Total estimated motor neuron numbers were obtained with the Optical Fractionator [29], using a computer-assisted microscope and the StereoInvestigator software (MicroBrightField, Williston, VT, USA). Cells were counted on the computer screen using an Optronics MicroFire digital camera mounted on a Nikon Eclipse E600 microscope. Ventral gray matter volume data from the reconstructed segments were also obtained with the associated data analysis software NeuroExplorer (MicroBrightField). One 30 μm-thick ChAT-reacted section every five and one Nissl-stained section every fifteen were reconstructed at P4; one ChAT-positive and Nissl-stained section every eighteen were reconstructed at P13; one 25 μm-thick Nissl-stained section every eighteen was reconstructed at E19. The guard zones were 3 μm, the counting frame size was 75 × 75 μm and the sampling grid size 100 × 100 μm. The soma size of ChAT^+^ motor neurons was also analyzed in P4 and P13 mice at C3-C5 levels.

***Spinalmotorneuronpoolanalysis***. Four cervical motor neuron pools (phrenic nucleus at C4, deltoid and biceps at C5-C6, forearm muscles at C8-T1 levels, medial Ax9 at C1-C8) were identified in serial Nissl-stained and ChAT-reacted sections [30] of P4 (n=3) and P13 (n=4) mice. Sections (at least 3 sections for each motor neuron pool considered) were photographed with AxioCam MRc5 camera connected to Axioplan 2 Imaging microscope (Zeiss, Oberkochen, Germany) and soma cross-sectional areas measured at 40X magnification using Axiovision 4.8 software (Zeiss). Only cell bodies clearly showing a nucleolus on the plane of the section were considered. Mean motor neuron areas were calculated for each pool, and size distribution histograms (expressed in percentage) were constructed by grouping cross-sectional areas in 100 µm^2^ bins. 

### Motor cortex analysis

Cortical sections at P4 (n=3 for each genotype) and P9 (WT, n=4; SMA, n=3) stained with Cresyl violet were analyzed using the Neurolucida software program (Microbrightfield Inc.). Motor cortex (relative to one hemisphere) was identified and cell counts were performed in layers II-III and V. One 20 μm-thick section every five was reconstructed in a cerebral segment corresponding to plates 29, 36 and 39 of the Franklin and Paxinos atlas [31], and counts were performed at 40X magnification. The density of neurons was obtained using Neurolucida Explorer for computer-aided microscopy (Microbrightfield Inc.). The total number of counted cells was divided by the measured motor cortex surface area (expressed in μm^2^), and then multiplied by the section thickness (20 μm) in order to obtain a density volume value. Soma size of layer II-III and V pyramidal neurons was also measured in the motor cortex by random sampling (WT mice n=3; SMA mice n=3). Neuronal profiles of at least 100 cells (with the nucleolus on the plane of the section and avoiding the glial cells, identified by their smaller size) were outlined with the Neurolucida software and data analyzed with Neurolucida Explorer.

### Astrogliosis quantification

GFAP immunoreactivity was also analyzed to evaluate astroglial activation. The density of GFAP^+^ immunofluorescent profiles was quantified both in brain and spinal cord by 2 separate observers. Four areas were selected: layers II-III and V in motor cortex (1.42 mm and 0.26 mm from bregma), spinal ventral and dorsal horns at C5, for each animal (WT mice n=3; SMA mice n=4, two sections for each animal) at P13. These areas were photographed at 40X magnification using AxioCam MRc5 connected to the Axioplan2 Imaging microscope and fluorescence intensity quantified, after subtraction of background, using the ImageJ-ProPlus software. The intensity of fluorescence was expressed in Arbitrary Units (AU) as mean ± SEM.

### Western Blot Analysis

Spinal cords were quickly removed from P4 and P13 WT mice after decapitation, then immediately frozen on dry ice and stored at -80° C until use. Samples were homogenized in buffer containing 20 mM Hepes (pH 7.4), 1 mM DTT, 0.1 mM PMSF and Complete (Roche, Indianapolis, IN, USA). Protein extracts were separated by 12 % SDS-PAGE, electroblotted onto nitrocellulose membranes, probed with goat polyclonal anti-ChAT antibody (Millipore: diluted 1:150 in 3% non-fat milk) and analyzed with chemiluminescence (ECL; Amersham Int., Little Chalfond, UK). A monoclonal antibody against actin was used as loading control (Millipore: diluted 1:4000 in 3% non-fat milk). 

### Statistics

The values from each animal were averaged and each genotype group (WT and SMA) compared to achieve the p-value by unpaired Student’s t-test, two tails. Data for Student’s t-test significance were performed in Microsoft Excel. To test the differences in area size-interval frequencies between the two experimental groups (WT and SMA) we applied the chi-square test, using a free web utility (http://www.quantpsy.org/chisq/chisq.htm). Data were expressed as mean ± SEM (standard error of the mean) and differences were considered significant when p ≤ 0.05.

## Results

### Time course of spinal motor neuron loss in SMA

**Figure 1:**
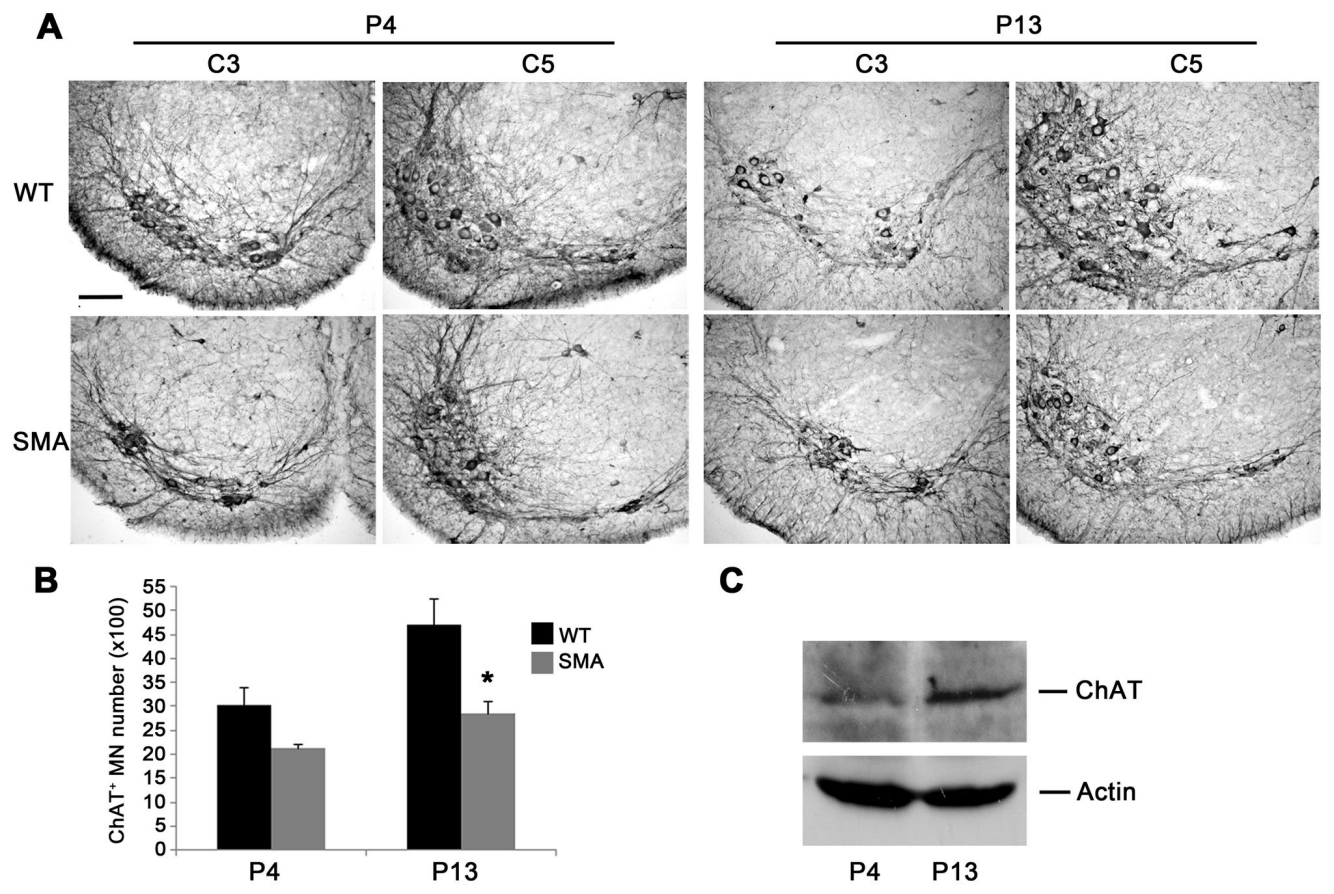
Stereological analysis of ChAT^+^ motor neurons. (**A**) Low-power images of ChAT immunoreacted spinal cord sections at ventral C3-C5 cervical levels from WT (upper) and SMA (lower) mice at pre- (P4, left) and late-symptomatic (P13, right) disease stages. Note that the C5 area of ChAT^+^ immunoreactivity was smaller in SMA than in WT mice at both P4 and P13. (**B**) Stereological analysis revealed that total motor neuron number was reduced at P4 (left) and significantly decreased at P13 (right) in SMA compared to WT mice (^*^p<0.05, t-test). (**C**) Western-blot analysis revealed increased ChAT expression between P4 and P13 in WT mice. Values are expressed as mean ± SEM. Scale bar: 100 µm.

To verify the onset and progression of spinal motor neuron loss, we first compared stereological counts on ChAT immunoreacted sections at cervical C3-C5 levels in SMA (-/-) *vs* WT (+/+) mice, at pre- (P4) and late- (P13) symptomatic stages. Even at low magnification (Figure 1A), the ventral horn area of ChAT^+^ motor neuron cell bodies and neuropil was smaller in SMA mice *vs* WT at both P4 and P13. The smaller size was more evident at C5, where motor neurons innervating proximal forelimb muscles were located. Stereological analysis revealed that motor neuron number was decreased at P4 (SMA 2117 ± 84 cells; WT 3024 ± 366 cells, p > 0.05, t-test; Figure 1B left) and significantly reduced at P13 (SMA 2863 ± 242 cells; WT 4705 ± 630 cells, ^*^p < 0.05; Figure 1B right). The apparently contradictory increase in ChAT^+^ motor neuron number at P13 *vs* P4 in both WT and SMA mice was explained as due to age-dependent maturation in ChAT expression by western blot experiments, demonstrating increased ChAT expression between P4 and P13 in the ventral spinal cord of WT mice (Figure 1C). Progression of spinal cord atrophy was also confirmed by volume analysis of the ventral C3-C5 segments (P4: 0.6 ± 0.02 mm^3^ in SMA *vs* 0.7 ± 0.04 mm^3^ in WT, p>0.05, t-test; P13: 0.8 ± 0.08 mm^3^ in SMA *vs* 1.3 ± 0.06 mm^3^ in WT, ^**^p<0.01).

**Figure 2:**
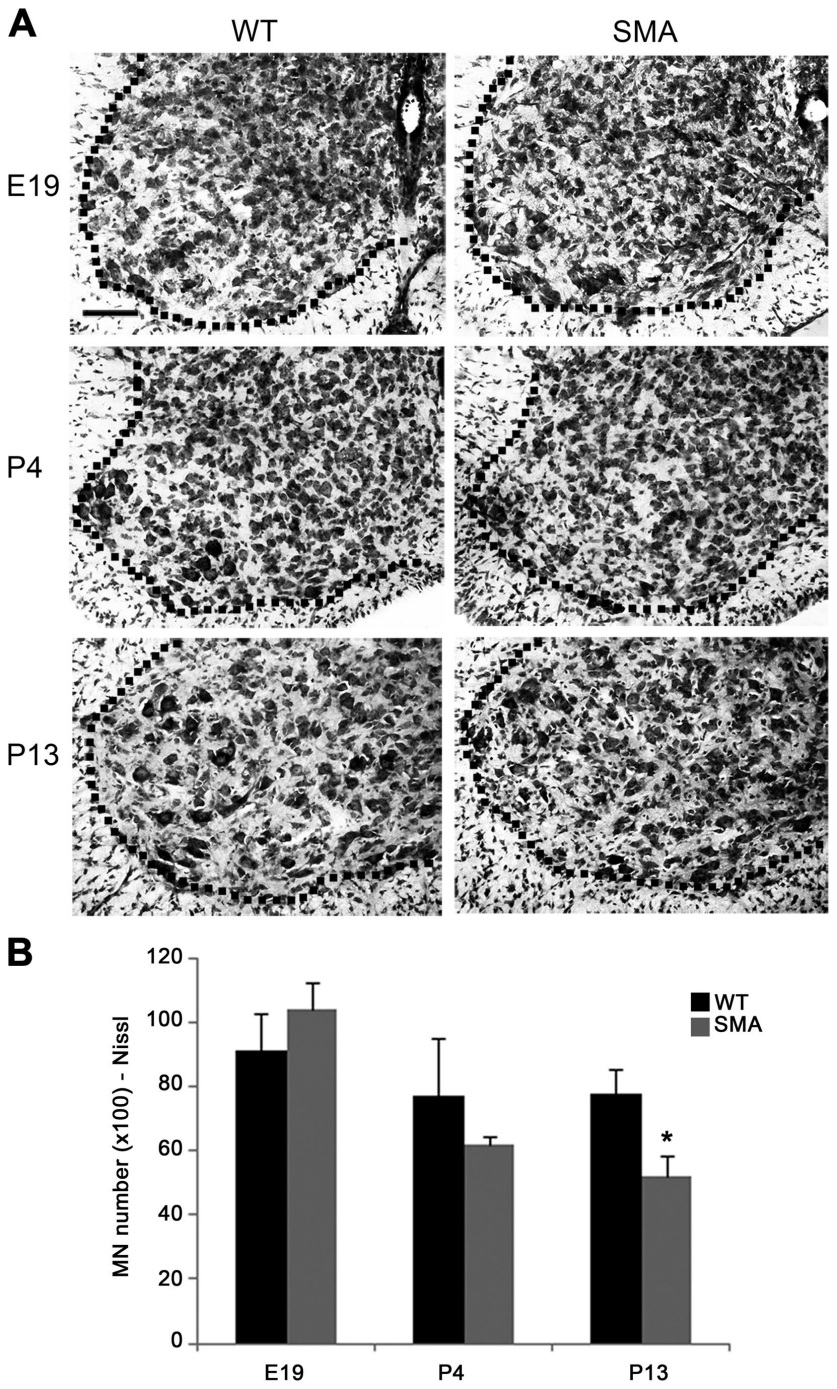
Stereological analysis of Nissl-stained motor neurons. (**A**) Representative Nissl-stained coronal sections of ventral C5 spinal cord from WT (left) and SMA (right) mice at prenatal (E19, upper), pre- (P4, middle) and late-symptomatic (P13, lower) disease stages. (**B**) No difference in the total cervical motor neuron number was evident at prenatal stages (E19, left), whereas a progressive reduction was evident post-natally (P4, middle), reaching statistical significance at late-symptomatic stages (P13, right: ^*^p<0.05, t-test). Values are expressed as mean ± SEM. Scale bar: 100 µm.

The reduction in motor neuron number at P4 could be related to early motor neuron loss but also due to impairment of motor neuron development during spinal cord ontogenesis. We therefore verified cervical motor neuron numbers in pre-natal (E19) and post-natal (P4 and P13) SMA mice and WT controls in Nissl-stained sections (Figure 2A), to avoid possible confounding bias from the age-dependent expression of ChAT. Stereological counts carried out in the whole cervical tract at E19 did not reveal differences in motor neuron number in SMA *vs* WT mice (Figure 2B). In keeping with data on ChAT^+^ motor neurons, progressive reduction of Nissl-stained motor neurons occurred during post-natal stages, becoming significant at P13, i.e., when motor neuron function was clearly impaired (P4: 6103 ± 264 motor neurons in SMA mice, 7703 ± 1774 in WT, p>0.05, t-test; P13: 5180 ± 657 motor neurons in SMA, 7798 ± 716 in WT, ^*^p< 0.05; Figure 2B).

### Loss of large motor neurons in SMA

**Figure 3:**
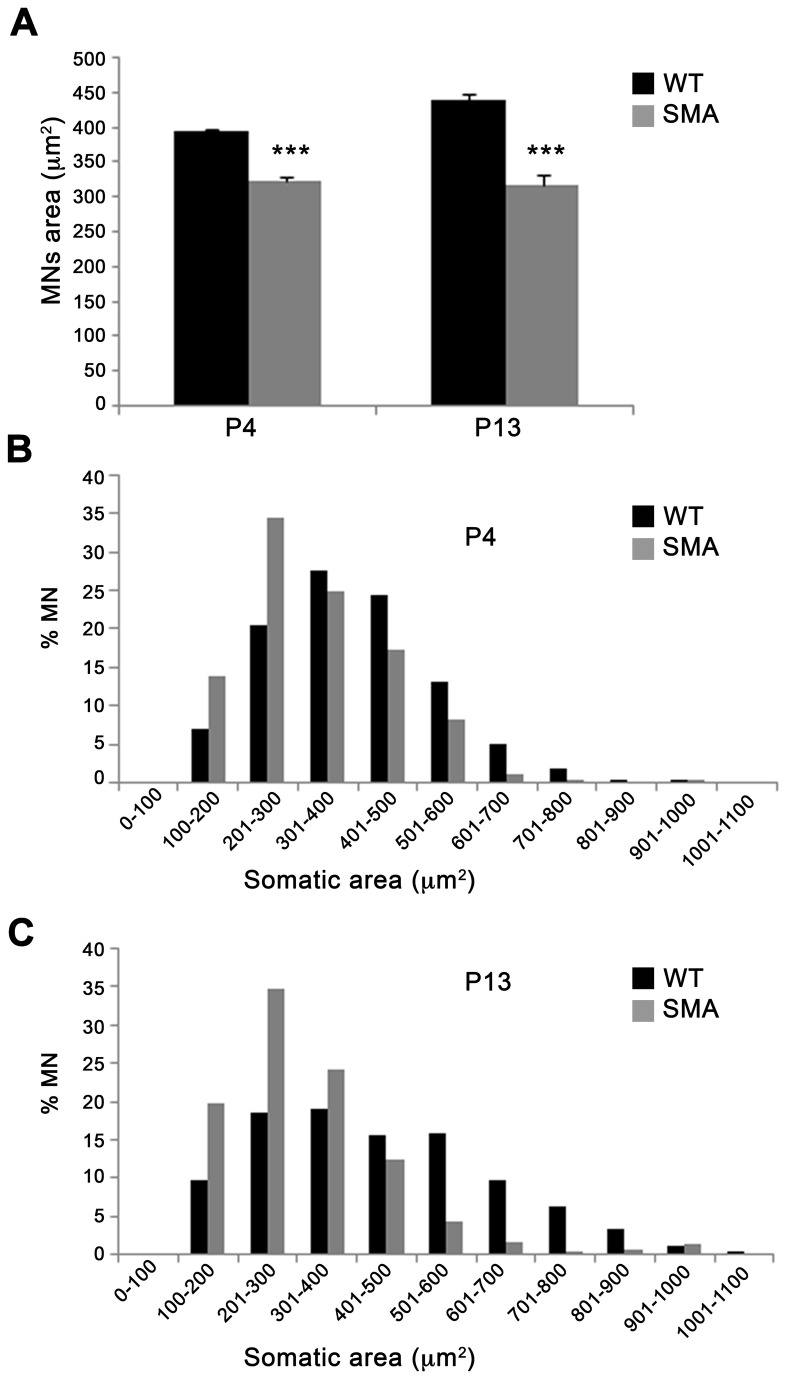
Large motor neurons are selectively affected in SMA. (**A**) Quantitative analysis at C3-C5 spinal levels revealed a significant reduction of mean motor neuron area in SMA *vs* WT mice at P4 (left) and P13 (right, ^***^p<0.001, t-test). (**B**, **C**) The analysis of the distribution of motor neuron areas (bins=100 µm^2^) revealed a significant shift to lower size in SMA *vs* WT mice at both P4 and P13 (^***^p<0.001, chi-square test between WT and SMA curves). Also note the reduction of motor neurons larger than 500 µm^2^ in SMA mice. Values are expressed as mean ± SEM.

The reduced ChAT^+^ neuropil area at P4, i.e., at a stage when motor neuron number was not significantly reduced, prodded us to measure the size of C3-C5 ChAT^+^ motor neurons. Motor neurons were significantly smaller in SMA *vs* WT mice at P4 and P13, indicating the possibility that motor neurons underwent atrophy before dying (P4: 324 ± 4.3 µm^2^ in SMA and 394.7 ± 3.8 µm^2^ in WT, ^***^p < 0.001, t-test; P13: 316.4 ± 15.3 µm^2^ in SMA and 438 ± 11.3 µm^2^ in WT, ^***^p < 0.001; Figure 3A). The distribution of motor neuron soma size was significantly shifted to the lower area size at P4 and more clearly at P13 (^***^p<0.001, chi-square test, at both ages; Figure 3B and C), and the percentage of motor neurons with cross-sectional area greater than 500 µm^2^ was reduced both at P4 (9.7 ± 0.8% *vs* 20.54 ± 1.2%, ^**^p<0.01, t-test) and P13 (8.7 ± 2% *vs* 37 ± 3%, ^***^p<0.001, t-test) in SMA *vs* WT mice. These data might indicate in ∆7 SMA mice either a more selective loss of larger motor neurons or a developmental failure leading to smaller motor neurons.

### Selective vulnerability of motor neurons innervating proximal and axial muscles

**Figure 4:**
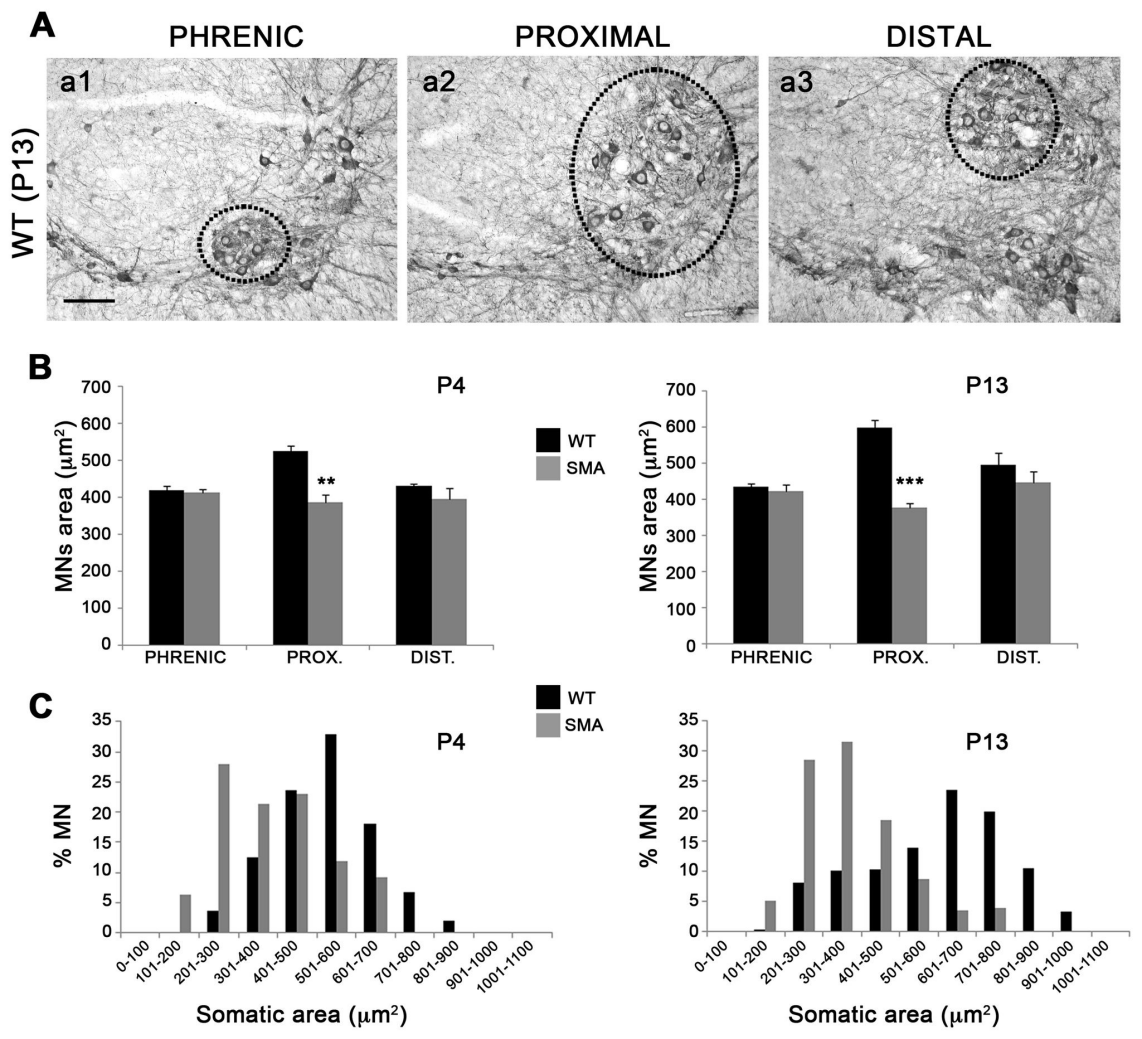
Selective loss of proximal motor neurons. (**A**) Representative ChAT reacted coronal sections of the cervical spinal cord illustrating 3 different motor neuron (MN) pools (hatched lines) in WT at P13: phrenic MNs at C3 level (a1), lateral MNs at C5-C6 innervating forelimb proximal muscles (a2), and lateral MNs at C8 innervating forelimb distal muscles (a3). (**B**) Quantitative analysis of mean areas of the 3 MN pools in SMA *vs* WT mice revealed a significant soma size reduction only in the MN pool innervating proximal forelimb muscles (left, P4: 386.3 ± 22.5 µm^2^ in SMA *vs* 526.5 ±15 µm^2^ in WT, ^**^p < 0.01, t-test; right, P13: 378 ± 13 µm^2^ in SMA *vs* 599.7 ± 20.7 µm^2^ in WT, ^***^p < 0.001). Mean area were unchanged in phrenic MNs (left) and not significantly reduced in MNs innervating distal muscles (right). (**C**) The analysis of soma size distribution of MNs innervating proximal muscles revealed a significant shift to lower size in SMA *vs* WT mice at P4 (left) and even more at P13 (right) (^***^p<0.001, chi-square test between WT and SMA curves). Values are expressed as mean ± SEM. Scale bar: 100 µm.

**Figure 5:**
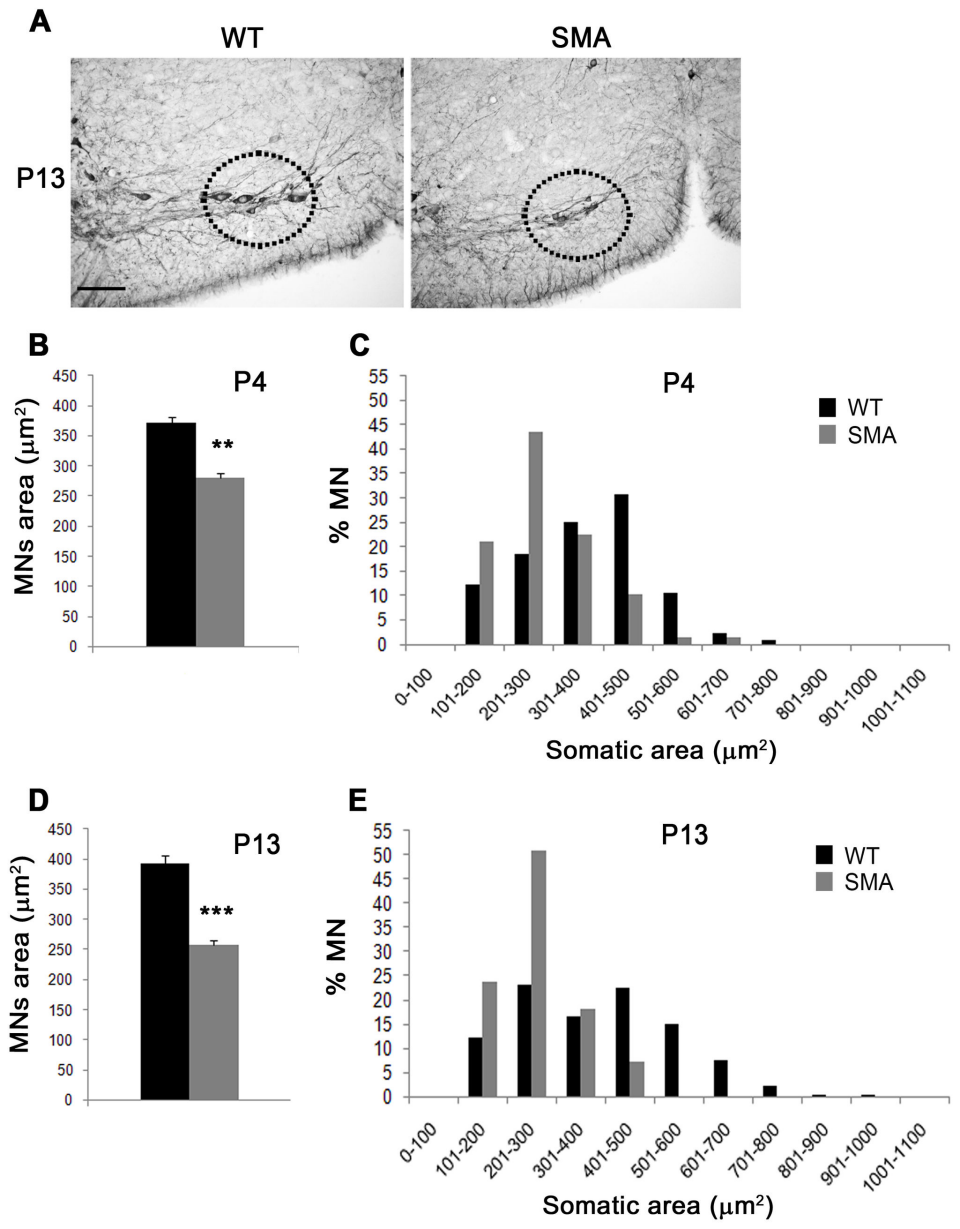
Selective loss of axial motor neurons. (**A**) Representative ChAT reacted coronal sections of the cervical spinal cord illustrating Ax9 medial motor neurons (hatched lines) innervating axial muscles in WT (left) and SMA (right) at P13. Note the smaller neuronal size in SMA vs WT mice. (**B**, **D**) The average cross-sectional area was significantly reduced in SMA mice at both P4 (**B**) and P13 (**D**) stages (P4: 280 ± 8 µm^2^ in SMA *vs* 371 ±10.5 µm^2^ in WT, ^**^p < 0.01, t-test; P13: 258 ± 7 µm^2^ in SMA *vs* 392 ± 14 µm^2^ in WT, ^***^p < 0.001). (**C**, **E**) The distribution of MN cross-sectional areas at both P4 (**C**) and P13 (**E**) showed a significant shift to lower size in SMA *vs* WT mice (^***^p<0.001, chi-square test between WT and SMA curves). Values are expressed as mean ± SEM. Scale bar: 100 µm.

Since different muscle groups are differently affected in SMA, we compared in WT *vs* SMA mice four specific motor neuron pools of the cervical region at P4 and P13: (i) phrenic motor neurons at C4, innervating the diaphragm, (ii) lateral motor neurons at C5-C6, innervating forelimb proximal muscles, (iii) lateral motor neurons at C8, innervating forelimb distal muscles, and (iv) medial Ax9 motor neurons at C1-C8, innervating axial muscles (see Figures 4A and 5A for representative motor neuron pools at P13). We found that mean somatic area in SMA *vs* WT mice was reduced in motor neurons innervating proximal forelimb and axial muscles only (Figures 4B and 5B, D). The reduction was already significant at P4 (^**^p<0.01, t-test; Figures 4B left and 5B) and even more evident at P13 (^***^p < 0.001; Figures 4B right and 5D). By contrast, mean area was unmodified in phrenic motor neurons and slightly but not significantly reduced in motor neurons innervating distal forearm muscles (Figure 4B). The distribution of soma size in motor neurons innervating proximal and axial muscles revealed a statistically significant shift to the lower area size at P4 and more clearly at P13 (^***^p<0.001 at both ages, chi-square test between SMA and WT curves; Figures 4C and 5C, E), further indicating either more selective vulnerability of large size motor neurons or developmental impairment in these motor neuron pools. By contrast, soma size distribution analysis of the two other motor neuron pools did not reveal evident changes (Figure S1).

### Loss of layer V pyramidal neuron of motor cortex

**Figure 6:**
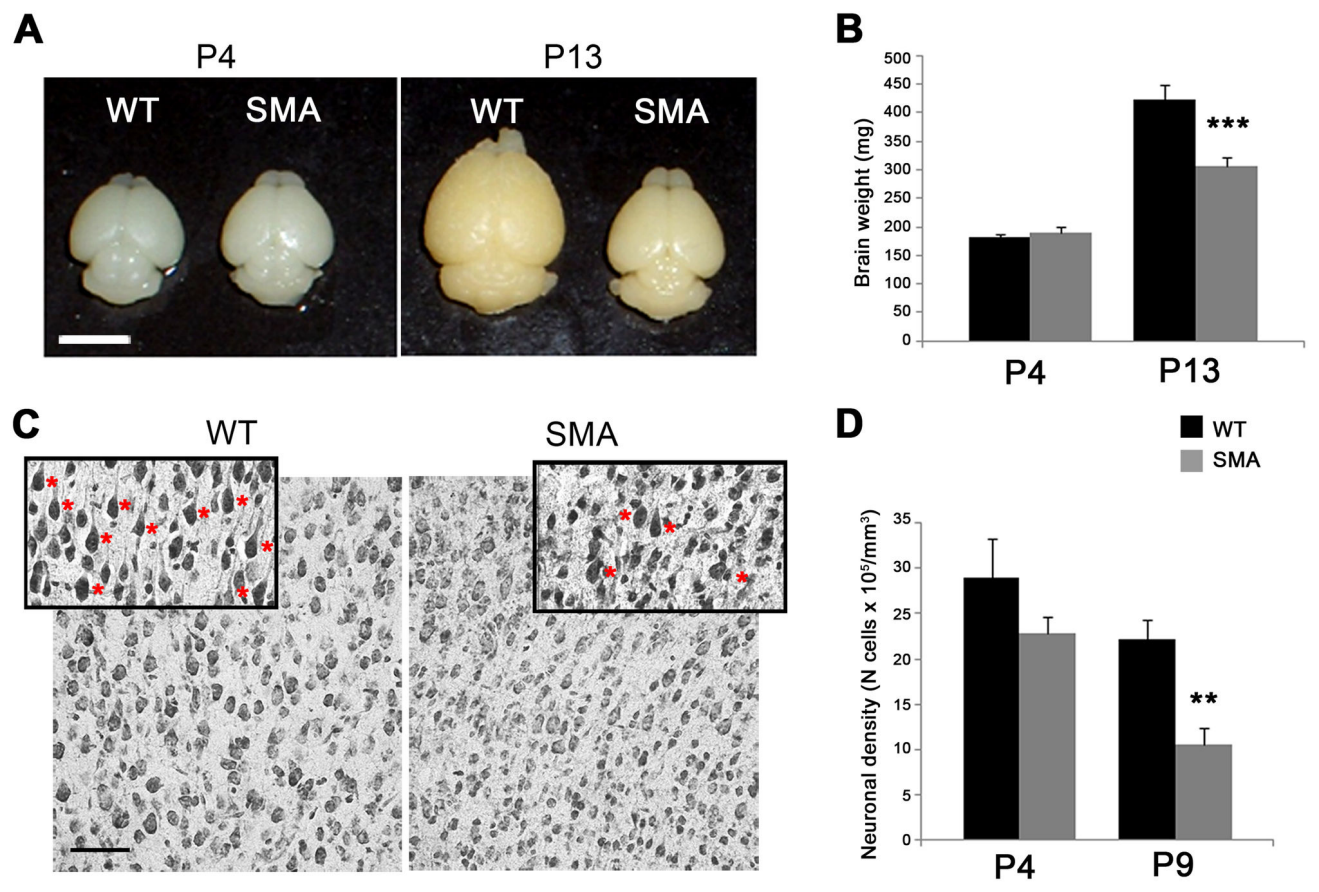
Loss of layer V pyramidal neurons of motor cortex. (**A**) Photographs of P4 and P13 mouse brains showing a clear reduction of brain size in SMA *vs* WT mice at P13 (right). No difference in size were observed at P4 (left). (**B**) Quantitative analysis of brain weight in SMA *vs* WT littermates at P4 and P13. A weight reduction of about 30% was observed at late-symptomatic (P13: ^***^p<0.001, t-test) but not at pre-symptomatic (P4) stages. (**C**) Nissl-stained brain sections illustrating reduced numbers of larger pyramidal neurons (red asterisks in the inset) in layer V of SMA mice *vs* WT controls. (**D**) Stereological counts of Nissl-stained brain sections revealing a significant 52% decrease (^**^p<0.01, t-test) of pyramidal neuron density in layer V of P9 SMA *vs* WT mice. The density of pyramidal neurons was already reduced at P4, without reaching statistical significance (p>0.05). Values are expressed as mean ± SEM. Scale bars: 5 mm in A; 50 µm in C.

We next investigated whether Smn deletion affected also the upper motor neuron compartment by analyzing brain morphology at pre- (P4) and symptomatic (P9 and P13) stages, with particular attention to layers II-III and V of the motor cortex. Whole brain comparison between SMA and WT mice revealed a clear reduction in size and weight at P13 (SMA 307.4 ± 13.4 mg *vs* WT 423 ± 25 mg, ^***^p < 0.001, t-test; Figure 6A right, 6B) but not at P4 (SMA 190.5 ± 8.3 mg *vs* WT 182.6 ± 4.6 mg, p > 0.05; Figure 6A left, 6B). Stereological counts performed on Nissl-stained coronal brain sections (Figure 6C) revealed that cell density of pyramidal neurons in layer V was slightly reduced in SMA *vs* WT mice at P4 (SMA 2278320 ± 179975 cells/mm^3^; WT 2896685 ± 435636 cells/mm^3^, p >0.05, t-test) and significantly decreased at P9 (SMA 1056858 ± 176559 cells/mm^3^; WT 2219833 ± 218528 cells/mm^3^, ^**^p < 0.01; Figure 6D). By contrast, cell density in layers II-III did not differ between SMA and WT mice at either stages (data not shown), suggesting that layer V neurons, i.e., long-projecting corticofugal neurons, were more selectively susceptible in SMA mice. At fully-symptomatic stages (P9), the mean area of pyramidal neurons was slightly but not significantly reduced in both layers II-III and V (Figure S2) and the soma size distribution analysis of both layers II-III and V neurons did not reveal evident changes (Figure S2).

### Glial activation in SMA mice

**Figure 7:**
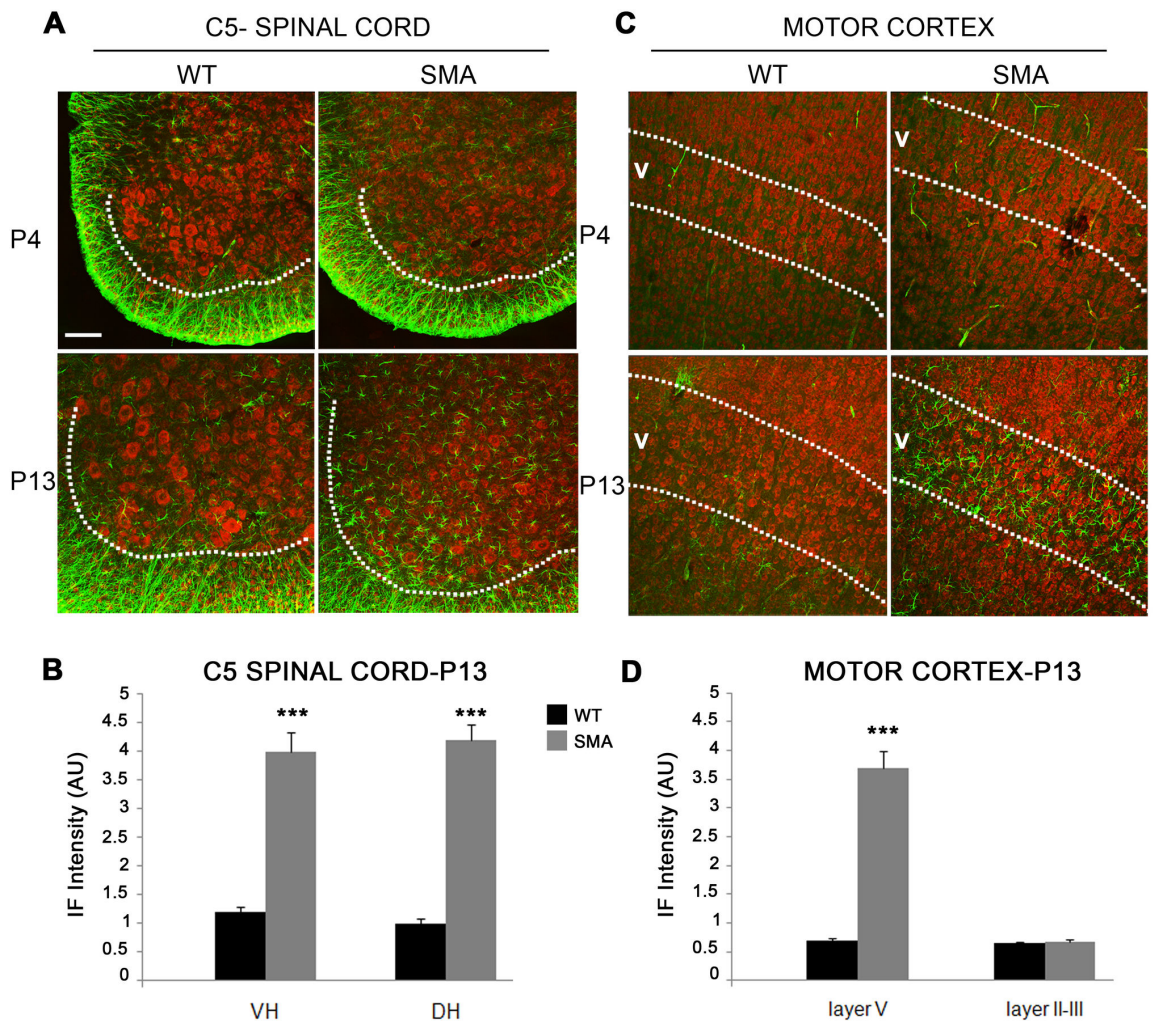
Glial activation in Δ7 SMA mice. Coronal sections from cervical spinal cord (**A**) and brain (**C**) of WT (left) and SMA mice (right) stained with Neurotrace (red) and GFAP-immunoreacted (green). Note glial activation in the spinal cord and cortical layer V at P13 (bottom right in A and C) but not at P4. (**B**, **D**) Quantification of fluorescence intensity (expressed in arbitrary units, AU) revealed a significantly increased glial activation in both ventral (VH) and dorsal horns (DH) of the spinal cord (**B**) and in cortical layer V but not layers II-III (D) of SMA mice at P13 (^***^p<0.001, t-test). Values are expressed as mean ± SEM. Scale bar: 100 µm.

We finally evaluated cerebral and spinal glial activation in sections reacted for GFAP immunofluorescence (IF) in SMA *vs* WT mice at P4 and P13 (Figure 7). GFAP^+^ IF signal did not differ between SMA and WT mice at P4 in either C5 spinal cord and cerebral motor cortex (Figure 7A and C, upper). By contrast, reactive gliosis was significantly increased in SMA *vs* WT mice at P13 in both spinal cord and cerebral cortex (Figure 7A and C, lower, 7B and D, ^***^p < 0.001, t-test). Interestingly, reactive gliosis was increased in both ventral and dorsal horns of the spinal cord (Figure 7B) but selectively in layer V (and not layers II-III) of the motor cortex (Figure 7D), supporting the selective neuronal loss in layer V described above. 

## Discussion

We have here investigated motor neuron loss in the spinal cord and cerebral motor cortex of ∆7 mice [17], the most widely used SMA model, using stereological quantification methods. We found progressive post-natal loss of spinal motor neurons, already at pre-symptomatic stages, and a higher vulnerability of larger motor neurons and those innervating proximal muscles. We also demonstrated a selective reduction of layer V pyramidal neurons in the cerebral motor cortex. Our data indicate therefore that, at least in this model, i) SMN loss is particularly critical for specific motor neuron pools; and ii) neuronal loss is not strictly selective for spinal motor neurons.

### Methodological considerations

Our stereological analysis was performed on spinal cords processed for IHC with the specific motor neuron marker ChAT, and Nissl staining. ChAT labels motor neurons selectively, but is age-dependent, since ChAT expression matures through development. In addition, ChAT does not identify early pathologic events in SMA, since the occurrence of chromatolitic, ChAT^+^ motor neurons can be observed only after birth in SMAI patients [32]. Nissl staining labels all cells from the earliest stages of development, allowing identifying motor neurons by their size and position, but it might exclude smaller motor neurons or, vice versa, include different neurons such as large spinocerebellar cells located in the ventral horns. Previous data, based on non-stereological analysis, provided different quantification of motor neuron loss in ∆7 mice, ranging from no loss to 25% loss in lumbar motor neurons at terminal stages [27,33]. The use of both methods, allowing the analysis of the time-course of motor neuron loss through development (by Nissl staining) and the precise differences in motor neuron number between WT and SMA mice (by ChAT staining), indicated clearly that progressive death of motor neurons is a relevant event taking place in the spinal cord of ∆7 mice.

### Motor neuron loss in the spinal cord

Indeed, the presence of comparable cervical motor neurons counts at E19 in both ∆7 SMA mice and WT indicated that motor neuron development in Δ7 mice was not grossly impaired by Smn deficiency. By contrast, the progressive reduction of motor neuron counts in SMA *vs* WT mice (30% motor neuron loss at P4 and further 10% occurring between P4 and P13) demonstrated a neurodegenerative process taking place in Smn-depleted motor neurons. These data also suggest that motor neuron death is more severe in early post-natal stages, followed by a period of relative stability characterized by minor motor neuron loss. The time course of motor neuron loss is in agreement with previous data reporting no further denervation of vulnerable neuromuscular junctions (NMJs) in ∆7 mice after P7 [34]. 

The precise evaluation of number and post-natal fate of motor neurons simply portrays the end-point but it does not describe the whole neurodegenerative process, since many subtle changes might affect the proper function of the motor unit before motor neuron loss. Recent studies in ∆7 SMA mice demonstrated altered Ca^2+^ homeostasis and morpho-functional abnormalities of the NMJs preceding motor neuron loss, and neurofilament accumulation in axons and terminals without wallerian degeneration, therefore suggesting that failure of NMJ maintenance and/or the proper crosstalk between axon terminals and muscle fibers could be the first step in the pathogenesis of SMA [27,35-37]. However, many other factors should be considered. Motor neurons in ∆7 mice are hyperexcitable, receive reduced central synapses and VGLUT1^+^ proprioceptive sensory inputs before motor neuron loss [38,39], and axonal swellings and muscular denervation were reported in embryonic severe and ∆7 SMA mice [34,40], clearly indicating more subtle motor unit damage well before the establishment of a clear motor phenotype. Altered neuromuscular transmission does not fully explain motor paralysis in SMA mice, and, even though axial muscles are more vulnerable and motor neuron death is more prominent in the medial than in the lateral motor column, neither muscle location nor fiber type (i.e., fast *vs* slow twitch) or motor unit phenotype (i.e., fast- *vs* delayed-synapsing) [41] could be considered as selective determinants of motor failure in SMA mice [34,36-38]. Careful muscle analysis in ∆7 SMA mice suggested the pathogenic relevance of neuromuscular development defects [42]. Apoptotic mechanisms can also play a major role in motor neuron death in SMA, as suggested by data from human post-mortem specimens of SMA type I patients [43,44] and animal models [45,46]. Finally, it should be remembered that failure in motor neuron migration was proposed as the key step to understand motor neuron loss and hence pathogenesis in human SMA [47].

All together, these data emphasize the complexity of SMA pathogenesis, which is likely related to multiple factors converging in affecting the integrity and function of the neuromuscular unit. Our data suggest that larger motor neurons and specific motor neuron pools (for axial and proximal forelimb muscles) in the cervical spinal cord are more vulnerable, either for selective loss or specific developmental impairment leading to motor neurons of smaller size. Motor neurons for axial and proximal muscles supply a large number of terminal motor units in a complex tridimensional fashion. Since SMN is likely involved in axonal transport of relevant molecules [12,48], this function might be more easily challenged in motor neurons with extensive axonal branching that support many different terminal motor units.

### Cortical changes

Even though it is generally reported in literature that SMA patients “only showed decreased numbers of anterior horn cells in the spinal cord, with no change in the cortical pyramidal cells, including the Betz cells, and no degeneration of the pyramidal tracts” [49], we have surprisingly detected a selective decrease in the number of large layer V pyramidal neurons in the motor cortex, indicating that also upper motor neurons degenerate/undergo atrophy in ∆7 SMA murine brain. By contrast, layer II-III neurons in the motor cortex did not show cell loss. The decrease in the density of large size layer V pyramids can be related to the loss of target lower motor neurons. It might be due to cell shrinkage, in absence of neuronal death, as described after corticospinal tract section [50], possibly for the lack of trophic support [51]. Even if the death of cortical motor neurons following lower motor neurons loss is unlikely, since only damage close to cortical neuron soma can cause cell death [52], it cannot be entirely ruled out since immature corticospinal neurons are strictly dependent on their target [53]. Indeed, cortical neuronal death was reported during prenatal brain development in severe SMA mice [54]. Clear abnormalities of brain morphology and development were reported post-natally in the same mouse model, in both neocortex and hippocampus [55]. Together with the latter data on hippocampus and the reduced sensory input to spinal motor neurons [39], our results emphasize that SMN deficiency deeply alters the development and fate of multiple neuronal subpopulations.

### Astrogliosis

Finally, we observed a marked gliosis both in the spinal cord and in the cerebral cortex of affected mice at P13, whose location matched that of motor neuron and layer V pyramidal cell loss. The astrocytic network plays an important role in synaptic function and plasticity by providing metabolic support to neurons and participating to the reuptake and release of neuro-transmitters [56,57] and it might be relevant in the pathogenesis of neurodegenerative diseases. Activated astrocytes can either assure a permissive substrate for axonal regeneration/sprouting and produce neurotrophins and anti-inflammatory cytokines, or favor motor neuron degeneration and death by releasing toxic molecules, inducing oxidative stress, and upregulating pro-inflammatory cytokines [58]. In this respect, intramuscular administration of adenoviral vectors expressing cardiotrophin-1 improved motor performance and increased survival in a SMA model [59], indicating that cytokines might modulate the neurodegenerative process in SMA. The present data are in agreement with the report of gliosis in the spinal cord of SMAI and SMAII patients [47], and support the idea that also neuroinflammation should be considered as a potential therapeutic target for tackling SMA.

### Conclusion

In summary, we show here the detrimental effect of reduced levels of SMN protein in lower and upper motor neurons and the time-course of their loss, and the selective involvement of motor neuron pools, extending our current knowledge on SMA neuropathological hallmarks. The pathogenesis of SMA is likely more complex than previously anticipated, involving neuronal and non-neuronal systems, as demonstrated by the report of cardiac muscle defects and glucose intolerance in SMA patients and mouse models [15,60,61]. Our findings should stimulate further analyses of the neuropathology and clinics of SMA. Different promising therapeutic approaches for SMA are now developing, and our study suggests that their target should not be directed to lower motor neurons only. Understanding properly the nature and progression of the anatomopathological manifestations of the disease can give useful suggestions on the timing of therapeutic interventions to prevent their occurrence.

## Supporting Information

Figure S1 **Soma size distribution of phrenic nucleus and motor pool innervating distal muscles.** Analysis of soma size distribution of phrenic MNs (**A**) and in MNs innervating distal muscles (**B**) did not reveal evident changes in SMA compared to WT mice both at P4 (left) and P13 (right) stages.
(TIF)

Figure S2 **Soma size analysis of cortical motor neurons in WT and SMA mice.** (**A**) Mean cross-sectional areas of cortical neurons in layers V and II-III at P9 revealed a slight, non-significant size reduction in SMA *vs* WT mice (layer V: 47.5 ± 5.2 µm^2^ in SMA *vs* 54.2 ± 5 µm^2^ in WT; layers II-III: 40 ± 2.7 µm^2^ in SMA *vs* 51.6 ± 9 µm^2^ in WT, p>0.05, t-test). (**B**-**C**) Area distribution analysis (20 µm^2^ bins) did not reveal significant changes between WT and SMA mice at P9 in both layers V (B) and II-III (**C**) (p>0.05, chi-square test between WT and SMA curves).
(TIF)
