# Supplementary figures and images for: Selective Vulnerability of Spinal and Cortical Motor Neuron Subpopulations in delta7 SMA Mice
Source: PLoS One. 2013 Dec 6;8(12):e82654. doi: 10.1371/journal.pone.0082654 (PMC3855775; doi:10.1371/journal.pone.0082654)

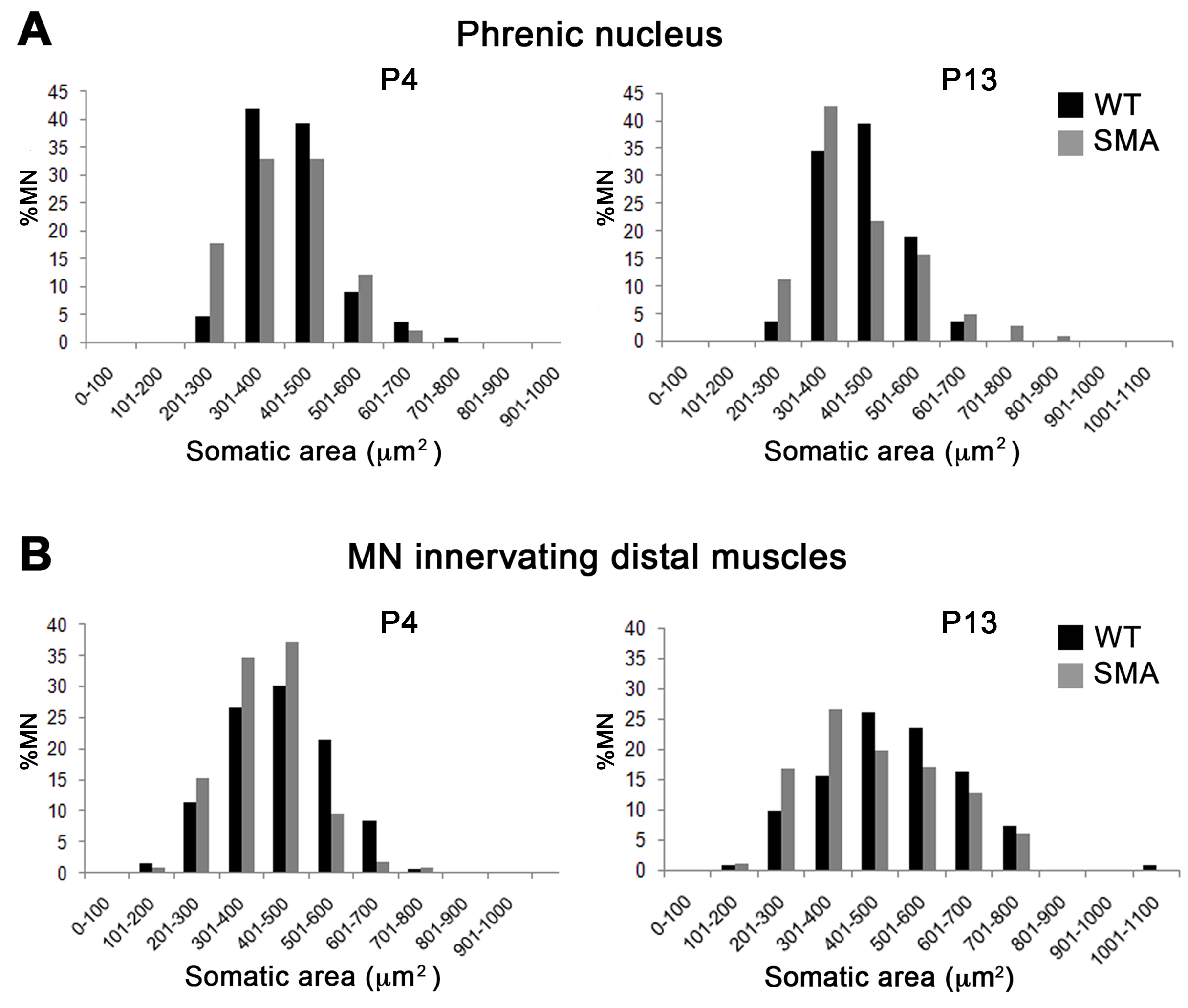

Supplement: Figure S1 — Soma size distribution of phrenic nucleus and motor pool innervating distal muscles. Analysis of soma size distribution of phrenic MNs (A) and in MNs innervating distal muscles (B) did not reveal evident changes in SMA compared to WT mice both at P4 (left) and P13 (right) stages. (TIF) [file pone.0082654.s001.tif]

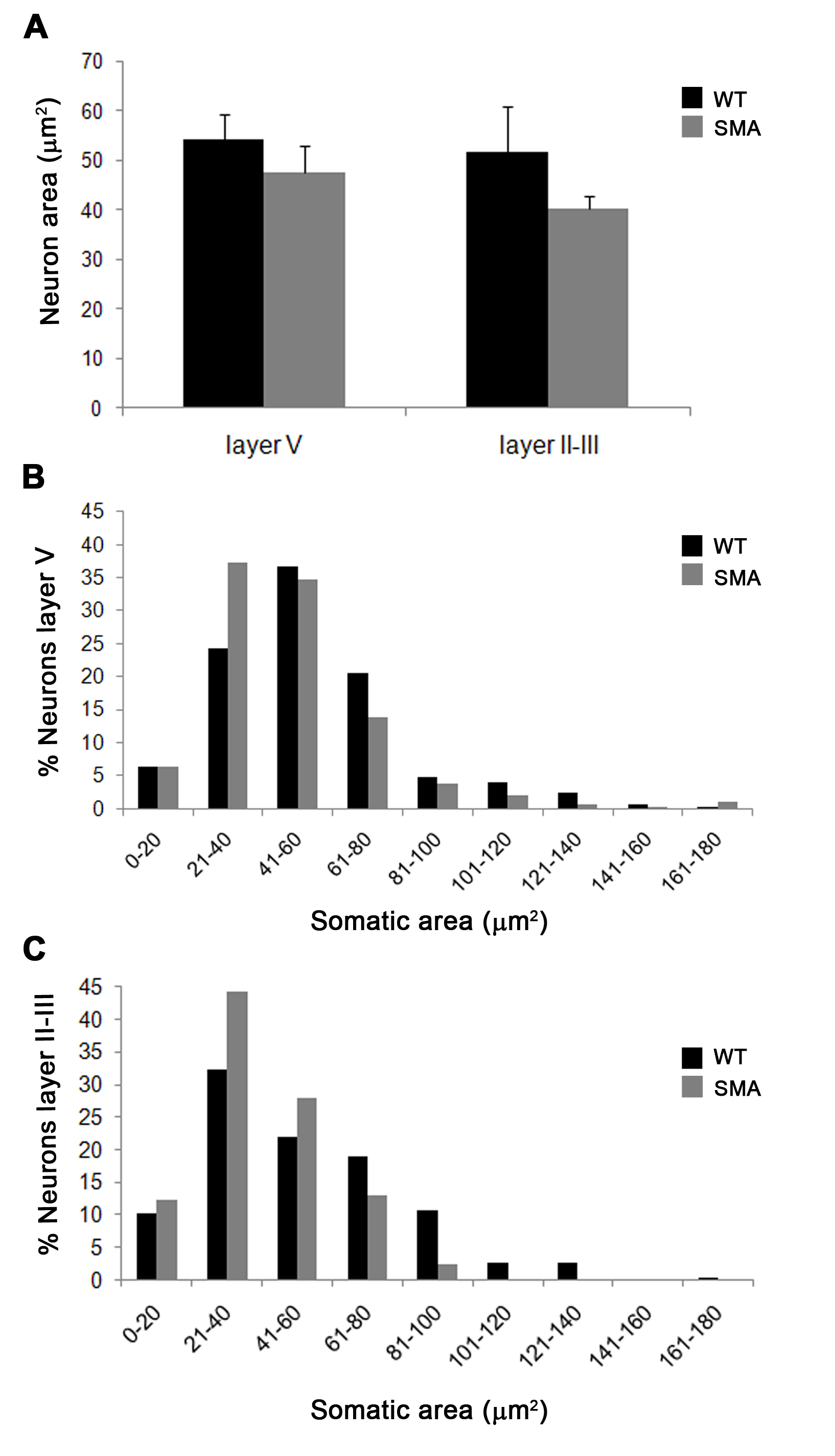

Supplement: Figure S2 — Soma size analysis of cortical motor neurons in WT and SMA mice. (A) Mean cross-sectional areas of cortical neurons in layers V and II-III at P9 revealed a slight, non-significant size reduction in SMA vs WT mice (layer V: 47.5 ± 5.2 µm2 in SMA vs 54.2 ± 5 µm2 in WT; layers II-III: 40 ± 2.7 µm2 in SMA vs 51.6 ± 9 µm2 in WT, p>0.05, t-test). (B-C) Area distribution analysis (20 µm2 bins) did not reveal significant changes between WT and SMA mice at P9 in both layers V (B) and II-III (C) (p>0.05, chi-square test between WT and SMA curves). (TIF) [file pone.0082654.s002.tif]
